# A comparison of Covid-19 early detection between convolutional neural networks and radiologists

**DOI:** 10.1186/s13244-022-01250-3

**Published:** 2022-07-28

**Authors:** Alberto Albiol, Francisco Albiol, Roberto Paredes, Juana María Plasencia-Martínez, Ana Blanco Barrio, José M. García Santos, Salvador Tortajada, Victoria M. González Montaño, Clara E. Rodríguez Godoy, Saray Fernández Gómez, Elena Oliver-Garcia, María de la Iglesia Vayá, Francisca L. Márquez Pérez, Juan I. Rayo Madrid

**Affiliations:** 1 ETSI Telecomunicación, iTeam Institute, Universitat Politècnica València, Camino de Vera S/N, 46022 València, Spain; 2 Instituto Física Corpuscular, National Research Council (CSIC)-Universitat València, València, Spain; 3 Instituto de Física Corpuscular IFIC (CSIC-UVEG), Madrid, Spain; 4 PRLHT Research Center, Universitat Politècnica de València, València, Spain; 5 Hospital General Universitario Morales Meseguer, Murcia, Spain; 6 Complejo Hospitalario Universitario de Badajoz, Badajoz, Spain; 7 Biomedical Imaging Mixed Unit, FISABIO-CIPF, Fundación para el Fomento de la Investigación Sanitaria y Biomédica de la Comunidad Valenciana, València, Spain; 8 Regional Ministry of Universal Health a Public Health in València, València, Spain

**Keywords:** Deep learning, Covid-19, Radiology

## Abstract

**Background:**

The role of chest radiography in COVID-19 disease has changed since the beginning of the pandemic from a diagnostic tool when microbiological resources were scarce to a different one focused on detecting and monitoring COVID-19 lung involvement. Using chest radiographs, early detection of the disease is still helpful in resource-poor environments. However, the sensitivity of a chest radiograph for diagnosing COVID-19 is modest, even for expert radiologists. In this paper, the performance of a deep learning algorithm on the first clinical encounter is evaluated and compared with a group of radiologists with different years of experience.

**Methods:**

The algorithm uses an ensemble of four deep convolutional networks, Ensemble4Covid, trained to detect COVID-19 on frontal chest radiographs. The algorithm was tested using images from the first clinical encounter of positive and negative cases. Its performance was compared with five radiologists on a smaller test subset of patients. The algorithm's performance was also validated using the public dataset COVIDx.

**Results:**

Compared to the consensus of five radiologists, the Ensemble4Covid model achieved an AUC of 0.85, whereas the radiologists achieved an AUC of 0.71. Compared with other state-of-the-art models, the performance of a single model of our ensemble achieved nonsignificant differences in the public dataset COVIDx.

**Conclusion:**

The results show that the use of images from the first clinical encounter significantly drops the detection performance of COVID-19. The performance of our Ensemble4Covid under these challenging conditions is considerably higher compared to a consensus of five radiologists. Artificial intelligence can be used for the fast diagnosis of COVID-19.

**Supplementary Information:**

The online version contains supplementary material available at 10.1186/s13244-022-01250-3.

## Key points

- The sensitivity of COVID-19 detection using conventional CXR depends on the evolution of the disease.
- Compared to a group of radiologists, AI achieves a higher sensitivity at the onset of the disease.
- This ability for early detection of COVID-19 could be applied in settings lacking resources, providing an immediate radiological report for each patient studied with CXR. Assistance with this tool could also be supplied by teleradiology in places where there is a lack of microbiological testing.

## Background

The emergence of the Coronavirus Disease 2019 (COVID-19) has dramatically changed the way of life of millions of people worldwide, putting public health at risk and overburdening health systems. The number of cases worldwide is over 70 million, and the number of direct deaths is around 4.7 million as of September 2021 [1]. Early detection of Covid-19 cases remains crucial to reducing transmission rates and adopting appropriate treatment measures [2].

The standard method for COVID-19 diagnosis is reverse transcription-polymerase chain reaction (RT-PCR) [3]. At the beginning of the pandemic, when RT-PCR tests were scarce, imaging tests were crucial for diagnosing COVID-19. However, though chest computed tomography (CT) [4] or radiograph [5, 6] have been relevant throughout the evolution of the pandemic, the initial focus on identifying COVID-19 through lung involvement has changed. At the same time, different laboratory tests became increasingly available. Currently, a chest radiograph (CXR) is the recommended imaging test for the initial assessment of patients with respiratory symptoms suspected to be caused by SARS-COV-2 infection [7]. The primary role is now to be a biomarker of a higher severity level of lung damage and a follow-up tool for that lung involvement over time, including the speed of lung spreading. The relevance of those clinical variables has motivated the development of different artificial intelligence algorithms for automated detection of COVID-19 chest involvement [8–11].

There are many previously reported results for COVID-19 detection using AI with chest radiographs. Initially, many studies were limited to small datasets or used publicly available images of variable quality [12, 13]. Still, other recent studies have used more extensive and more consistent datasets producing more reliable results [10]. An earlier diagnosis of COVID-19 would allow us to anticipate care at a higher therapeutic level [14, 15]. Still, the predictive power of CXR in the initial stages, either for radiologists or for an AI algorithm, is lower. We hypothesize that our AI algorithm can still detect radiological signs of COVID-19 in chest CXRs performed in the initial stages of the disease, with a performance that could be competitive against radiologists. Although in developed countries, COVID-19 diagnosis does not currently depend on imaging techniques as it did during the first stage of the pandemic, a reliable AI algorithm could be of substantial help for world places where analytic resources are systematically scarce. However, in the post-pandemic future, it could be helpful to differentiate between COVID-19 in the very early stages of the disease and other diseases evolving with similar clinical features for highly demanded and overloaded emergency and radiology departments. That algorithm would be a relevant clinical tool for triaging respiratory patients. Therefore, based on CXR images, our objective was to compare the performance for detecting COVID-19 of an automated AI algorithm with the radiologists' interpretation. But, unlike other related algorithms, our CXR images correspond to the first one performed on patients with suspected COVID-19 pneumonia for both positive and negative cases. On the other hand, in our research, negative cases were patients who never showed positive in any RT-PCR test during the same period of the study reducing the possibility of bias compared to previous works in which non-COVID-19 cases were selected from pre-pandemic era patients [9, 16]

## Materials and methods

The checklist for Artificial Intelligence in Medical Imaging (CLAIM) has been added as an Additional file 1 [17] as suggested by Roberts et al. in [18].

### Data

Our retrospective study included patients from the publicly available BIMCV-COVID19 + [19] and BIMCV-COVID19- [20] datasets for positive and negative patients, respectively. The source of this data is the Medical Imaging Databank in Valencian Region (Spain) (BIMCV) which includes 23 hospitals from 18 health departments.

The datasets contained CXR images of COVID-19/non-COVID-19 patients along with polymerase chain reaction (RT-PCR), immunoglobulin G (IgG), and immunoglobulin M (IgM) diagnostic antibody tests and radiographic reports. The CXR images were acquired using radiological equipment from 20 different vendors, ensuring large variability in the dataset. Patients were consecutively recruited between April and June 2020 (period of observation) who met the following inclusion criteria: (a) having an RT-PCR and/or IgM or IgG serological test for clinical suspicion of SARS-COV-2 infection, and (b) having a first portable or standing chest X-ray performed less than one week from the date of the microbiological or serological test. Patients under 18 years of age were excluded. Our gold standard test to discriminate between patients with and without acute SARS-CoV-2 infection was RT-PCR; however, given the limitations of the test and in-line with recommendations proposed in [21] serological tests (Ig) were also used. Therefore, patients with at least one positive RT-PCR, IgM, or IgG test during the observation period were classified as positive for SARS-COV-2 infection (hereafter "positive"). Patients with negative results in all serological or microbiological tests performed during the observation period were classified as patients without SARS-COV-2 infection (hereafter "negative"). A patient could have several studies and diagnostic tests with different outcomes during this period.

### Datasets

**Fig. 1:**
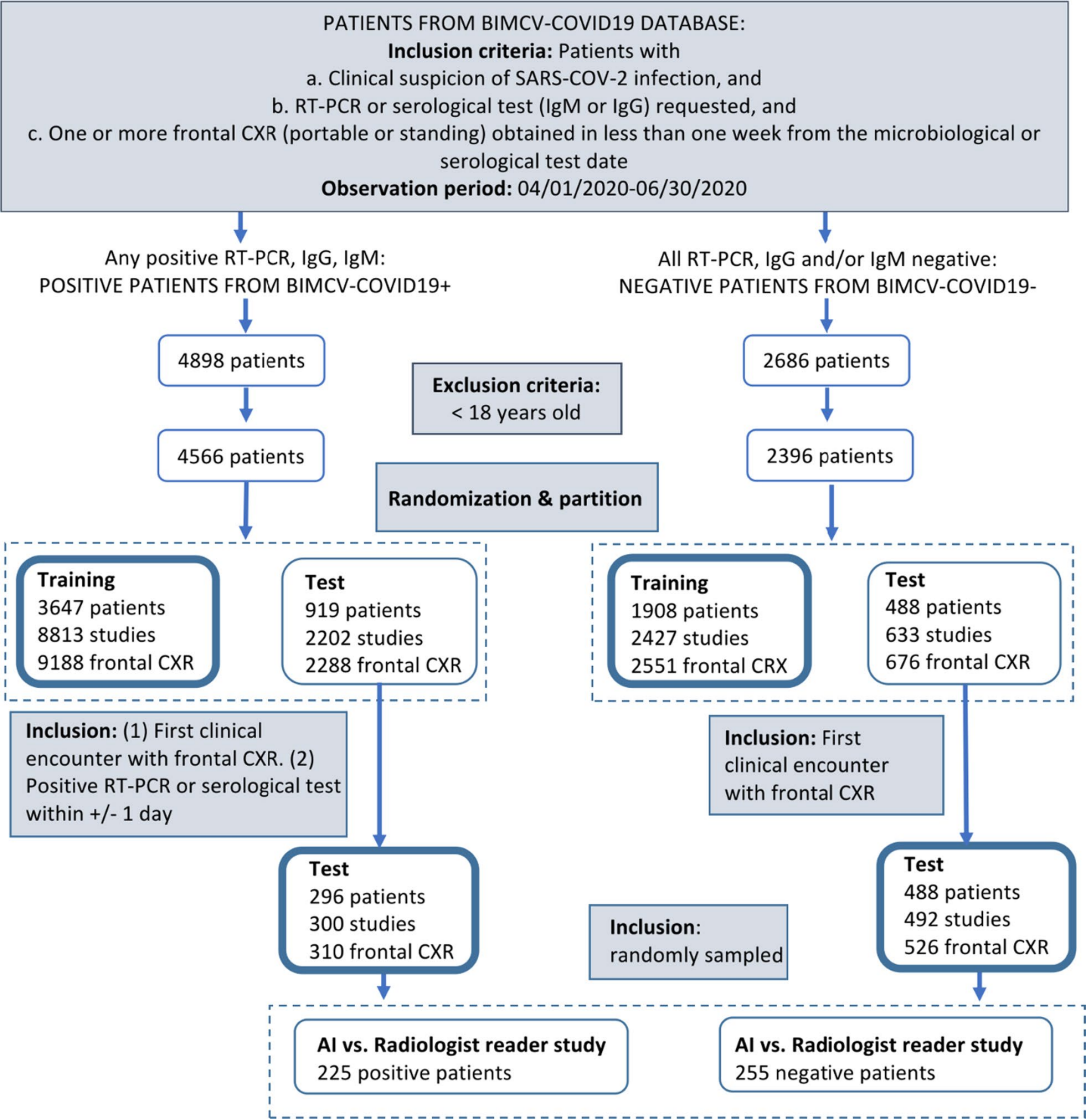
Flowchart for data curation and data partition

The study included 6,962 patients (4,566 positive and 2,396 negative) after excluding patients under 18 years old. A random split was carried out to get a training set independent from the test set. The training set included 3,647 positive patients (9,188 frontal CXR) and 1,908 negative patients (2,551 frontal CXR). The independent test set included the remaining patients who were a) 310 patients positive for SARS-CoV-2 infection whose CXR images met the requirements of being from the first clinical encounter and belonged to patients with a positive test within plus/minus one day, and b) 527 negative cases whose images corresponded to the first clinical encounter. It is important to emphasize that only the images from the first clinical encounter were retained for the test set since we were concerned with the early diagnosis of COVID-19. Figure 1 shows the detailed breakdown of the data. Notice that the number of frontal CXR is higher than the number of patients because one patient might have undergone several different clinical studies on the first day, and a clinical study may contain several frontal CXR. Notice that all frontal CXRs from all the clinical encounters were included in the training set. This setting increases the number of available training images, which is critical when training deep neural network models.

Finally, a subset of 225 positive and 255 negative studies was randomly sampled from the test set for radiologists' evaluation.

A detailed demographic description of the training and test datasets is presented in Table 1. The table shows no significant age and gender deviations between training and test groups and between positive and negative cases. The higher number of radiographs for the positive training patients is caused by the follow-up studies made on this particular group. Notice that this is not the case for the positive test patients because only the images from the first clinical encounter are used for evaluation. A factor that could bias the result is the different quality of the images captured from either mobile or fixed devices. Table 1 also shows the fraction of images obtained using mobile devices, which is similar for the distinct groups. Finally, the table also shows a higher incidence rate for the male group (*p* < 0.05), which is consistent with other previous studies [22].

**Table 1:** Detailed dataset description

		Training/validation			Test		
	Overall	Total	Covid19-pos	Covid19-neg	Total	Covid19-pos	Covid19-neg
Num patients	6339	5555	3647	1908	784	296	488
Age (mean ± std)	63 ± 18	63 ± 18	62 ± 17	63 ± 18	62 ± 18	62 ± 17	62 ± 19
Age (quantiles)	64 (38–86)	64 (38–86)	64 (40–86)	64 (37–87)	64 (38–86)	64 (38–83)	64 (38–87)
Female sex	3188 (50%)	2761(50%)	1754(48%)	1007(53%)	427 (54%)	151 (51%)	276 (56%)
AP, PA Chest radiographs per patient	1 (1–4)	1 (1–4)	2 (1–5)	1 (1–1)	1 (1–1)	1 (1–1)	1 (1–1)
Num. images		12,599	9855	2744	946	347	599
Num. images acquired with a mobile device		3699 (29%)	2970 (30%)	729 (27%)	230 (24%)	78 (22%)	152 (25%)

Age variables are presented as the mean and standard deviation and also using 10% and 90% quantiles. For the number of female cases, absolute and percentage values are shown. Quantiles are also used for the number of chest radiographs per patient

### Deep learning model

Like other works in the literature, our approach is based on the use of deep convolutional neural networks that are available as pre-trained models [23] to apply transfer learning techniques. Four different well-known convolutional architectures pre-trained with the ImageNet dataset [24] were used. In particular, the ResNet50 [25], Densenet121 [26], InceptionV3 [27] and InceptionResNetV2 [28] were chosen.

Before feeding the images into the model, the images are preprocessed by normalizing the pixel intensities using the maximum pixel value of each image and resizing them to a fixed resolution of 256 × 256 pixels. A convolutional layer with three channels at the beginning of the network was added to adapt these architectures to the gray-level CXR images. This layer serves as a pseudo-color conversion to fit the pre-trained input layer that expects color images. Moreover, a GlobalAveragePooling [29] and a Dense layer were added to the top of the pre-trained network with a sigmoid activation function to provide a score.

After all these modifications, the network was finally optimized by minimizing the binary cross-entropy using an Adam optimizer, learning rate annealing, and a batch size of 24 samples. The optimization was performed over a fixed number of 50 epochs with a learning rate of 0.001 for the first 25 epochs and 0.0001 for the last epochs. This network was fully trained end-to-end, including all the pre-trained network weights, using the Keras library [30].

A critical issue for training convolutional neural networks is choosing suitable data augmentation policies for the problem. In this sense, the RandAugment technique [31] was explored with the following $K = 9$ policies: AutoContrast, Equalize, Rotate, Contrast, Brightness, Sharpness, Cutout, TranslateX,and TranslateY. Moreover, RandAugment requires to specify two main parameters: $N$, the number of policies applied simultaneously, and $M$, the strength of these augmentations. RandAugment always selects a transformation with a uniform probability of $1/K$. Therefore, given N transformations for a training image, RandAugment may apply $K^{N}$ different policies. Different values for the $N \in \left\{ {4,5,6} \right\}$ and $M \in \left\{ {25,30} \right\}$ parameters were used, being 30 the maximum strength defined for each augmentation. In total, different combinations of 4 topologies, 3 values of $N$ and 2 values of $M$, were used to train a total number of 24 models. The possible values of the $N$ and $M$ were selected using a validation set extracted from the training set following an 80/20 partition. To this end, the area under the ROC curve (AUC) over the validation set was chosen as the metric to optimize.

### Comparison with radiologists

Five radiologists with 1, 3, 6, 12, and 14 years of experience, and recent intensive experience with COVID-19 CXR interpretation, provided evaluations to benchmark the model's performance using a smaller subset of 225 positive and 255 negative test patients, as shown in Fig. 1.

Radiologists were blinded to any clinical characteristics and had access to the complete radiologic study (i.e., radiologists could review both frontal and lateral projections). Radiologists provided an overall subjective interpretation of COVID-19 probability by using several labels following the COVID-19 BSTI reporting templates [32]: 1: Normal, 2: probable COVID-19, 3: Indeterminate for COVID-19, and 4: Other Non-COVID-19 abnormalities. The agreement among radiologists was measured using Fleiss' kappa statistic, which is a generalization of Cohen's kappa for multiple raters and ratings [33]. We tested the null hypothesis ($\alpha$ = 5%) that the underlying value of kappa is 0, which suggests an agreement by chance.

A three-point COVID-19 scoring system was derived using the radiologists' labels:

- Score 0 for the Normal and other Non-COVID-19 abnormalities labels.
- Score 1 for the intermediate probability of COVID-19.
- Score 2 for the high probability of COVID-19.

The radiologists' scores were given a threshold at intermediate values between the three scores (between 0 and 1 and between 1 and 2) to get two distinct operating points of sensitivity and specificity. A consensus radiologist interpretation was also obtained by averaging the scores of individual radiologists.

### Comparison with state-of-the-art AI algorithm using a public dataset

The AUC performance may change depending on the criteria used to build the training and test datasets. Hence, we have validated our training methodology using one of the most extensive open access benchmarks, COVID-19 datasets: the COVIDx dataset [12], for an objective comparison with other state-of-the-art methods. The COVIDx dataset comprises 13,975 CXR images across 13,870 patient cases. The COVIDx dataset has many COVID-19 positive samples obtained from five different publicly available data repositories of variable quality. The dataset has three other classes: normal, pneumonia, and COVID-19. Following the methodology of Wang et al. [12], the performance of a single model using the Densenet121 architecture was evaluated using the sensitivity and the positive predictive value using the same dataset partitions and training the model from scratch for a fair comparison. Confidence intervals were obtained using 2,000 bootstrap samples [34].

## Results

### Performance of deep learning model

Table 2 summarizes the best results over the test set for each different neural network architecture. The RandAugment was applied with the best parameters N and M obtained using a validation subset for each topology. The best result was obtained for the Densenet121 model with *N* = 4 and *M*  = 30 with an AUC of 0.845. A linear ensemble of these four models, named as *Ensemble4Covid*, achieved a final AUC of 0.856. The threshold value over the scores used to obtain the different metrics shown in the table is the one that maximizes the Youden index [35] in the *Ensemble4Covid* ROC curve (maximizes the sum of sensitivity and specificity).

**Table 2:** Results over the 836 test images for the different architectures and RandAugments parameters

Neural Net	*N*	*M*	AUC	Sensitivity (%)	Specificity (%)	TP	TN	FP	FN	Accuracy (%)
ResNet50	6	25	0.833	75	77	233	407	119	77	76.5
Densenet121	4	30	0.845	76	78	237	414	112	73	77.8
InceptionV3	4	30	0.830	80	73	250	382	144	60	75.6
InceptionResNetV2	4	30	0.822	74	76	229	400	126	81	75.2
Ensemble4Covid			0.856	78	81	242	425	101	68	79.8

The threshold over the scores used to obtain the sensitivity, specificity, true positives (TP), true negatives (TN), false positives (FP), and false negatives (FN) shown in the table is the one that maximizes the Youden index

**Fig. 2:**
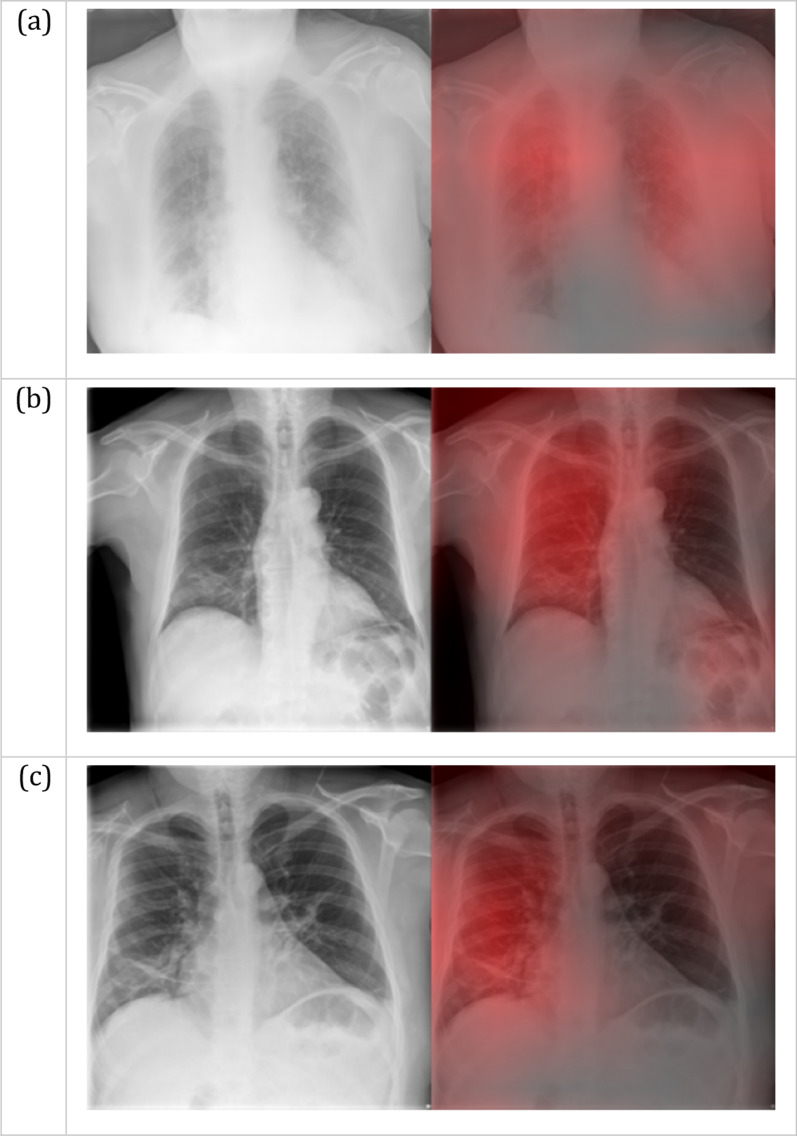
Grad-CAM heatmaps for True Positive COVID-19 predictions. The red intensity on the right images indicates the image regions that are important for the positive prediction. These regions correspond to patchy consolidations and ground-glass opacities, with a peripheral, bilateral distribution (**a**) or a mid and lower zone predominance on the right lung (**b**, **c**)

**Fig. 3:**
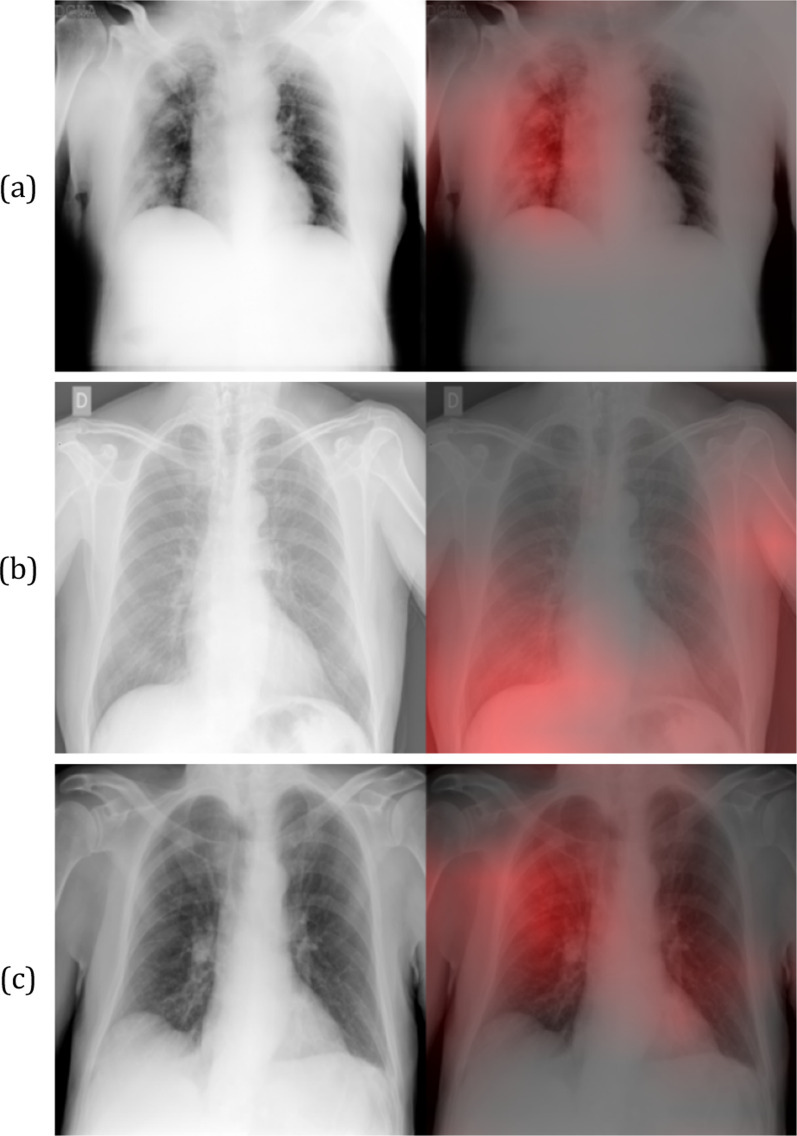
Grad-CAM heatmaps for False Positive COVID-19 predictions. The red intensity of the images on the right indicates the important regions for a false positive prediction. These regions correspond to multiple consolidations with no zone predominance in the right lung (**a**), a unilateral right lower lung opacity and the contralateral soft tissues in the axillary region (**b**), superimposition of the scapula in the right lung periphery along with the left lower lobe vessels behind the heart (**c**)

Figures 2 and 3 show three representative examples of true positive and false positive cases. A Grad-CAM heatmap [36] that highlights the feature importance is shown for each image. The Grad-CAM algorithm shows where the network is looking for a particular class. For this reason, only examples of positive predictions are displayed.

Table 3 displays the AUC results for different test groups using the *Ensemble4Covid* model. The performance was better in men (AUC 0.88 vs. 0.83) and slightly lower in patients younger than 40 years (AUC 0.82 vs. 0.86).

**Table 3:** Performance of Ensemble4Covid for different age and sex groups over the 836 test images

	Age group			Sex group	
	< 40	[40–60]	> 60	Male	Female
Num. patients	102	245	437	357	427
Num. images	109	257	470	381	455
AUC	0.82 (0.73–0.90)	0.86 (0.81–0.90)	0.86 (0.83–0.90)	0.88 (0.85–0.92)	0.83 (0.79–0.86)

Values in parenthesis are 95% confidence intervals

### Comparison with radiologists

A comparison between the radiologists and the ensemble neural network on the radiologists' test set (225 positive and 255 negative COVID-19 patients) is provided in Table 4. The agreement among radiologists using the Fleiss' Kappa test is significantly above chance ($\kappa = 0.35 \pm 0.05$, $\alpha = 0.05$). The table shows the specificity and sensitivity for each decision threshold. The sensitivity of the *Ensemble4Covid* model is provided for each mean specificity value.

**Table 4:** Radiologists’ specificity and sensitivity results for each threshold

Reader	Threshold	Specificity	Sensitivity	*Ensemble4Covid* sensitivity
Reader 1	High	98% (96–99%)	20% (14–25%)	31% (21–41%)
	Low	86% (82–90%)	35% (28–41%)	66% (58–76%)
Reader 2	High	96% (94–98%)	19% (14–24%)	41% (33–48%)
	Low	87% (83–91%)	32% (27–39%)	65% (55–73%)
Reader 3	High	98% (96–99%)	15% (10–20%)	31% (21–41%)
	Low	88% (84–92%)	25% (19–31%)	62% (53–71%)
Reader 4	High	86% (81–90%)	51% (44–58%)	66% (58–76%)
	Low	70% (64–75%)	61% (55–68%)	85% (79–89%)
Reader 5	High	82% (77–86%)	56% (50–62%)	76% (67–82%)
	Low	67% (61–73%)	69% (63–75%)	85% (81–90%)

The sensitivity of the Ensemble4Covid model for the same mean specificity is also displayed. Values in parenthesis are 95% confidence intervals

**Fig. 4:**
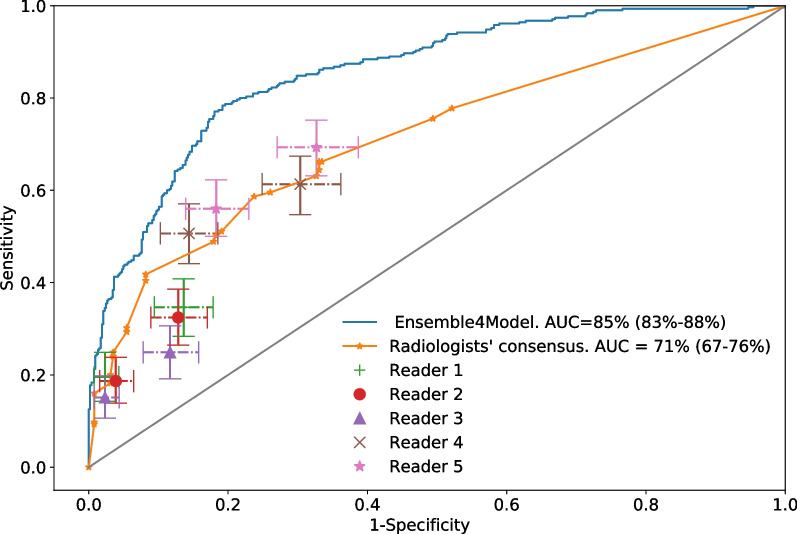
Sensitivity and specificity for each radiologist with 95% confidence intervals with the ROC curves for the Ensemble4Model (with 95% confidence bands) and the five radiologists' consensus. Each reader shows two operating points depending on the threshold over the scores

A consensus obtained by averaging the scores of the radiologists was obtained. Figure 4 compares the ROC curves of the *Ensemble4Covid* model and the five radiologists' agreement. It also displays the results for each radiologist. The five radiologists' consensus achieved an AUC of 0.71.

### Comparison with state-of-the-art artificial intelligence models

Table 5 displays the sensitivity and positive predictive value (PPV) of a single Densenet121 model compared with the COVID-Net results reported in Wang et al. [12]. The results show no significant statistical difference between the results presented in [12] and a trained Densenet121 model using the COVIDx dataset with the methodology described in the Materials and Methods section. The sensitivity achieved by the AI methods in the COVIDx dataset is much higher than the results achieved by the radiologists and our *Ensemble4Covid* method on our dataset (Table 3). This result indicates that the performance of the algorithms may be affected by the difficulty of the particular dataset and the established standard.

**Table 5:** Results on the COVIDx dataset

	Class	Densenet121	COVID-net
Sensitivity	Normal	0.94 (0.89–0.98)	0.95
	Non-COVID-19	0.94 (0.89–0.98)	0.94
	COVID-19	0.89 (0.82–0.94)	0.91
PPV	Normal	0.92 (0.86–0.97)	0.905
	Non-COVID-19	0.88 (0.81–0.93)	0.913
	COVID-19	0.98 (0.95–1)	0.989

Values in parenthesis are 95% confidence intervals obtained using 2000 bootstrap samples

## Discussion

Early detection of COVID-19 remains crucial, both for isolation and reduction of infections and for enabling patient monitoring and avoiding a fatal evolution [37]. CXR is not currently a suitable test for the early detection of COVID-19 disease [38]. However, CXR is an inexpensive, widely available, and highly requested test (especially in emergency departments) for any patient with rapid onset respiratory symptoms [39].

Our AI tool applied to CXR has reached a sensitivity value of 85% (79–89%) to detect COVID-19 for a specificity value of 70% (64–75%), higher than a group of five radiologists regardless of their experience in the CXR technique within an emergency environment.

Applying this tool systematically to all CXR performed in any health center would allow detection of the disease early without other tests [40]. Likewise, we have experienced continuous outbreaks of new variants whose severity, infectious potential, and symptoms are initially uncertain and need to be intensively investigated for weeks or months. In this context, the RT-PCR test may be less sensitive [41].

Moreover, if presented in an atypical manner, both clinical suspicion and the request for microbiological tests would be delayed. But whatever the clinical setting was, in the face of chest symptoms, CXR is usually carried out by default and before any microbiological tests, thus being COVID-19 potentially detectable through the AI tool.

On the other hand, in settings, countries, or situations with limited resources, it can be challenging to get the result of an RT-PCR test in a reasonable time when an early diagnosis is needed for patient-placement decisions. The AI algorithm systematically applied to CXR in this context could be a handy screening tool [38]. This assessment could also be carried out using teleradiology if the appropriate infrastructure was available.

The sensitivity of the *Ensemble4Covid* method is lower than that of other previously published algorithms [10, 12]. But in our research, the AI was evaluated just with the first radiological exam when pulmonary findings are less conspicuous than at a more advanced stage of the disease. However, we believe that our results are worth considering despite that apparent worse sensitivity because the validation conditions used in this research are closer to a real application scenario, where an early diagnosis is required. The advantage of AI may lie in its ability to identify changes in medical images before they become visible to the human eye. That would be the case with COVID-19 patients before abnormal lung densities were conspicuous in CXR.

Nevertheless, we have to recognize some limitations in our results. First, we do not know whether AI detects pneumonia or other abnormalities because no lung parenchymal segmentation was performed in training. These abnormalities may include pathology at different levels, e.g., cardiovascular and even extrapulmonary findings, such as data indicative of male sex or obesity, related to a worse prognosis of COVID-19 [42, 43].

This issue could make our AI vs. radiologist diagnostic performance unreliable and explain the significant difference in favor of AI. If this is the case, the comparison of sensitivities and specificities between AI and radiologists would be limited. But, in the end, the tool would still improve COVID-19 detection, as other publications with similar methodology have highlighted [44], especially for less experienced radiologists.

Accordingly, even if the AI performance was based on detecting extrapulmonary features indicative of vulnerability to COVID-19, it could also be suitable for clinical decision-making. As a result, an AI result suggesting COVID-19 could recommend closer clinical and radiological follow-up in patients with negative microbiological tests or radiological reports in the disease's early days. Future research will further elaborate on the findings that the tool is considering to reach its verdict.

Second, the software has been trained and tested in patients from the same centers. To determine if our results can be generalized, future studies will try to validate them using CXR performed by different radiological equipment in diverse sociodemographic populations and with different disease prevalence than those of the sample we used for training the algorithm [45]. However, the AUC parameter chosen to determine the tool diagnostic performance would vary minimally when applied in populations with a different disease prevalence.

A third limitation is that only CXR findings have been used in our analysis, without demographic or lab data that could improve the model's capability. The radiologist's assessment was also carried out under non-normal conditions to avoid biases in the pure interpretation of images, i.e., without clinical-analytical information.

A fourth limitation is that the tool's contribution to radiologists in clinical practice has not been assessed either. Therefore, we cannot rule out that the higher sensitivity of the AI could fade away in usual working environments, regardless of whether our data seemed to support that the AI improves the results even for radiologists with a higher level of expertise. This limitation could be overcome by prospectively testing the tool in an actual clinical situation.

The fifth limitation is that although the sample of positive and negative patients is similar in terms of age (Table 1), recruitment period (Fig. 1), and clinical suspicion of COVID-19, precise information on the symptoms and signs for COVID-19 suspicion and requested additional tests were lacking and may also have overestimated the performance of the AI tool. Our sixth limitation, inherent to the reference standard, as mentioned several times before, is that a negative RT-PCR result does not necessarily imply the absence of disease, and misclassifications may have occurred. We believe that using serological tests and microbiological tests has increased classification accuracy. Still, we do not rule out possible false negatives for SARS-CoV-2 infection in those patients who underwent only the RT-PCR test, especially if it was only once.

And our final limitation is that this study has demonstrated a high rate of false-positive results, especially in low-prevalent COVID-19 environments, up to 30% according to our specificity data. As a result, the number of RT-PCR or other microbiology or serological tests might be increased. However, the AI algorithm might increase its accuracy if additional information on patients' characteristics could be included.

In short, the preliminary results of this project suggest that this AI algorithm can be used for early detection of COVID-19 from the first conventional CXR performed at the onset of disease, overtaking the radiologists’ performance. The generalizability of our results, the reasons the algorithm bases its decisions, and the added value that it may bring to routine patient care will be elucidated in future work.

## Supplementary Information

**Additional file 1**. Checklist for Artificial Intelligence in Medical Imaging (CLAIM).

## Data Availability

The data is available on the BIMCV webpage https://bimcv.cipf.es/bimcv-projects/bimcv-covid19/#1590859488150-148be708-c3f3. The models, results, and list of images used for training, test, and comparison with radiologists are available at https://github.com/alalbiol/Covid-19-early-detection.

## Abbreviations

AI: Artificial intelligence
AUC: Area under the ROC curve
BIMCV: Medical Imaging Databank of Valencian Region
BIMCV-COVID19−: Dataset for negative patients
BIMCV-COVID19+: Dataset for positive patients
COVID-19: Coronavirus disease 2019
CT: Computed tomography
CXR: Chest radiograph
Grad-CAM: Gradient-weighted class activation mapping
IgG: Immunoglobulin G test
IgM: Immunoglobulin M test
PPV: Positive predictive value
ROC: Receiver operating characteristic
RT-PCR: Reverse transcription polymerase chain reaction
