# Correction: A comparison of Covid-19 early detection between convolutional neural networks and radiologists

**DOI:** 10.1186/s13244-022-01314-4

**Published:** 2022-10-20

**Authors:** Alberto Albiol, Francisco Albiol, Roberto Paredes, Juana María Plasencia-Martínez, Ana Blanco Barrio, José M. García Santos, Salvador Tortajada, Victoria M. González Montaño, Clara E. Rodríguez Godoy, Saray Fernández Gómez, Elena Oliver-Garcia, María de la Iglesia Vayá, Francisca L. Márquez Pérez, Juan I. Rayo Madrid

**Affiliations:** 1 ETSI Telecomunicación, iTeam Institute, Universitat Politècnica València, Camino de Vera S/N, 46022 València, Spain; 2 Instituto Física Corpuscular, National Research Council (CSIC)-Universitat València, València, Spain; 3 Instituto de Física Corpuscular IFIC (CSIC-UVEG), Madrid, Spain; 4 PRLHT Research Center, Universitat Politècnica de València, València, Spain; 5 Hospital General Universitario Morales Meseguer, Murcia, Spain; 6 Complejo Hospitalario Universitario de Badajoz, Badajoz, Spain; 7 Biomedical Imaging Mixed Unit, FISABIO-CIPF, Fundación para el Fomento de la Investigación Sanitaria y Biomédica de la Comunidad Valenciana, València, Spain; 8 Regional Ministry of Universal Health a Public Health in València, València, Spain

## Correction to: Insights into Imaging (2022) 13:122 https://doi.org/10.1186/s13244-022-01250-3

The original article [1] erroneously affiliated co-author, Ana Blanco Barrio to institution #6; this has since been corrected to affiliate Ana Blanco Barrio to institution #5.
