# Supplementary material for: A comparison of Covid-19 early detection between convolutional neural networks and radiologists
Source: Insights Imaging. 2022 Jul 28;13:122. doi: 10.1186/s13244-022-01250-3 (PMC9330942; doi:10.1186/s13244-022-01250-3)
Supplement: Supplementary file 1 — Additional file 1. Checklist for Artificial Intelligence in Medical Imaging (CLAIM). [file 13244_2022_1250_MOESM1_ESM.pdf]

CLAIM checklist

This document contains the CLAIM quality checklist as proposed in “Common pitfalls and recommendations for using machine learning to detect and prognosticate for COVID-19 using chest radiographs and CT scans” by M. Roberts et al. published in Nature Machine Intelligence, March 2021.

Section/Topic No. Item

TITLE or ABSTRACT

1 Identification as a study of AI methodology, specifying the category of technology used (eg, deep learning). **YES.**

ABSTRACT

2 Structured summary of study design, methods, results, and conclusions. **YES.**

INTRODUCTION

3 Scientific and clinical background, including the intended use and clinical role of the AI approach. **YES.**

4 Study objectives and hypotheses. **YES.**

METHODS

Study Design

5 Prospective or retrospective study. **YES.**

6 Study goal, such as model creation, exploratory study, feasibility study, noninferiority trial. **Not applicable.**

Data

7 Data sources **YES.**

8 Eligibility criteria: how, where, and when potentially eligible participants or studies were identified (eg, symptoms, results from previous tests, inclusion in registry, patient-care setting, location, dates). **YES.**

9 Data preprocessing steps. **YES.**

10 Selection of data subsets, if applicable. **YES.**

11 Definitions of data elements, with references to common data elements**. YES.**

12 De-identification methods. **YES.**

13 How missing data were handled. **Not applicable.**

Ground Truth

14 Definition of ground truth reference standard, in sufficient detail to allow replication. **YES.**

15 Rationale for choosing the reference standard (if alternatives exist) buscar ref. **YES.**

16 Source of ground truth annotations; qualifications and preparation of annotators **YES.**

17 Annotation tools. **Not applicable.**

18 Measurement of inter- and intrarater variability; methods to mitigate variability and/or resolve discrepancies. **YES**.

Data Partitions

19 Intended sample size and how it was determined. **NO.**

20 How data were assigned to partitions; specify proportions. **YES.**

21 Level at which partitions are disjoint (eg, image, study, patient, institution). **YES.**

Model

22 Detailed description of model, including inputs, outputs, all intermediate layers and connections. **YES**.

23 Software libraries, frameworks, and packages. **YES.**

24 Initialization of model parameters (eg, randomization, transfer learning). **YES**

Training

25 Details of training approach, including data augmentation, hyperparameters, number of models trained. **YES.**

26 Method of selecting the final model. **YES.**

27 Ensembling techniques, if applicable. **YES.**

Evaluation

28 Metrics of model performance. **YES**

29 Statistical measures of significance and uncertainty (eg, confidence intervals). **YES**

30 Robustness or sensitivity analysis. **NO.**

31 Methods for explainability or interpretability (eg, saliency maps) and how they were validated. **YES**

32 Validation or testing on external data. **YES.**

RESULTS

Data

33 Flow of participants or cases, using a diagram to indicate inclusion and exclusion. **YES.**

34 Demographic and clinical characteristics of cases in each partition. **YES.**

Model performance

35 Performance metrics for optimal model(s) on all data partitions. **YES.**

36 Estimates of diagnostic accuracy and their precision (such as 95% confidence intervals). **YES.**

37 Failure analysis of incorrectly classified cases. **YES**.

DISCUSSION

38 Study limitations, including potential bias, statistical uncertainty, and generalizability. **YES.**

39 Implications for practice, including the intended use and/or clinical role. **YES.**

OTHER INFORMATION

40 Registration number and name of registry. **Not applicable.**

41 Where the full study protocol can be accessed. **YES.**

42 Sources of funding and other support; role of funders. **YES.**
